# The Wisconsin Assessment of the Social and Built Environment (WASABE): a multi-dimensional objective audit instrument for examining neighborhood effects on health

**DOI:** 10.1186/1471-2458-14-1165

**Published:** 2014-11-13

**Authors:** Kristen C Malecki, Corinne D Engelman, Paul E Peppard, F Javier Nieto, Maggie L Grabow, Milena Bernardinello, Erin Bailey, Andrew J Bersch, Matthew C Walsh, Justin Y Lo, Ana Martinez-Donate

**Affiliations:** Department of Population Health Sciences, University of Wisconsin-Madison School of Medicine and Public Health, 610 N. Walnut Street, Madison, WI 53726 USA

**Keywords:** Neighborhoods, Built environment, Social environment, Population health, Measurement, Urban, Rural, Chronic disease, Prevention, Physical activity, Methods, Audit tool

## Abstract

**Background:**

Growing evidence suggests that mixed methods approaches to measuring neighborhood effects on health are needed. The Wisconsin Assessment of the Social and Built Environment (WASABE) is an objective audit tool designed as an addition to a statewide household-based health examination survey, the Survey of the Health of Wisconsin (SHOW), to objectively measure participant’s neighborhoods.

**Methods:**

This paper describes the development and implementation of the WASABE and examines the instrument’s ability to capture a range of social and built environment features in urban and rural communities. A systematic literature review and formative research were used to create the tool. Inter-rater reliability parameters across items were calculated. Prevalence and density of features were estimated for strata formed according to several sociodemographic and urbanicity factors.

**Results:**

The tool is highly reliable with over 81% of 115 derived items having percent agreement above 95%. It captured variance in neighborhood features in for a diverse sample of SHOW participants. Sidewalk density in neighborhoods surrounding households of participants living at less than 100% of the poverty level was 67% (95% confidence interval, 55-80%) compared to 34% (25-44%) for those living at greater than 400% of the poverty level. Walking and biking trails were present in 29% (19-39%) of participant buffer in urban areas compared to only 7% (2-12%) in rural communities. Significant environmental differences were also observed for white versus non-white, high versus low income, and college graduates versus individuals with lower level of education.

**Conclusions:**

The WASABE has strong inter-rater reliability and validity properties. It builds on previous work to provide a rigorous and standardized method for systematically gathering objective built and social environmental data in a number of geographic settings. Findings illustrate the complex milieu of built environment features found in participants neighborhoods and have relevance for future research, policy, and community engagement purposes.

**Electronic supplementary material:**

The online version of this article (doi:10.1186/1471-2458-14-1165) contains supplementary material, which is available to authorized users.

## Background

Understanding how the neighborhoods and communities in which we live influence health has important implications for future policy development and program planning. Research over the last decade highlights important and potentially modifiable neighborhood-level factors associated with health effects including metabolic disorders, obesity, depression, cardiovascular disease, diabetes, and cancer [1–4]. There is growing recognition and interest in understanding how social and built environment features affect a broader set of health determinants including sleep quality, overall wellness, and mental health outcomes [2, 4–8]. Despite this growing body of research, there is a paucity of information regarding the mechanisms by which such environmental features promote health, in part due to a lack of systematic methods and standardized tools for measuring neighborhood environments and features across diverse geographies including urban and rural areas [9, 10].

Increasingly, it is understood that a single approach to measuring neighborhood environments is insufficient to capture the breadth of environmental determinants of health and how they interact [5, 9–16]. Audit tools using standardized observation of social and built environments by field surveyors have emerged as one source of data to complement and improve the reliability and validity of measures available through Geographic Information Systems (GIS) or self-report survey data [9, 11–23]. While limited population-based studies have been conducted, few have included direct observations or audit instruments in their study designs. This may be in part due to limited feasibility and absence of protocols for data collection in diverse communities. The majority of prior studies using audit tools were designed specifically for active living research or have focused on narrowly-defined study populations (e.g., urban, elderly) [9, 10].

The Wisconsin Assessment of the Social and Built Environment (WASABE) was designed by an interdisciplinary team of researchers at the University of Wisconsin-Madison (UW) to systematically measure built and social environment features characterizing the neighborhoods of participants of an ongoing health examination survey, the Survey of the Health of Wisconsin (SHOW). We expected that integration of an objective audit tool into the SHOW program would identify differences in built environment features across diverse communities in Wisconsin and add to existing measures of neighborhood perceptions or extant Geographical Information System (GIS)-based measures [24]. This paper describes the development and implementation of the WASABE and presents reliability and validity data for this tool.

## Methods

### Context

Details on the design of the overall SHOW program (the parent study for the WASABE), including sampling scheme, have been described elsewhere [25]. Briefly, SHOW is a statewide household-based examination survey including a personal interview, a self-administered questionnaire, and a physical exam. The data are collected based on a social determinants of health model and include information on a wide-variety of health measures and health determinants. A two-stage stratified cluster sampling approach is employed to ensure that participants are recruited from all regions of the state and across diverse socio-demographic sub-groups. The UW-Madison Health Sciences Institutional Review Board approved all SHOW protocols and informed consent documents (protocol # H-2007-0261). Access to instruments, manuals, and codebooks can be found on the SHOW website at (http://www.show.wisc.edu).

WASABE development began with formative research including a systematic review of the literature, consultation with subject matter experts, and establishment of a scientific working group. Existing instruments that could be adapted for use in the diverse geographic landscapes and adhere to data collection protocols in SHOW were identified. After the tools and methods were outlined, several rounds of piloting and field-testing prior to final protocol development and implementation took place.

### Instrument development

The overall goal of the WASABE is to provide audit data on neighborhood-level physical features and social factors, emphasizing those related to physical activity and other health behaviors. The WASABE data were designed to complement SHOWs self-report data and existing GIS-based measures. Core concepts were drawn from previous active living surveys such as the Systematic Pedestrian and Cycling Environmental Scan, the Walking Suitability Assessment Form, the Analytic Audit Tool, the St. Louis Active Neighborhood Checklist, Irvine-Minnesota Inventory, and the Pedestrian Environment Scan [14–16, 18, 19, 26, 27]. A review of the literature linking built environment data to health behaviors and outcomes, particularly related to physical activity, was also conducted. The formative process of reviewing these surveys and associated literature led to the identification of five primary domains that were used to guide instrument development (neighborhood characteristics, transportation environment, destinations/land use, social environment, and street connectivity) [9, 17, 19, 28, 29]. Table 1 provides definitions and examples of features included in each domain and outcomes examined in previous research within each of the domains. The final four-page instrument [available in Additional file 1) includes 153 items. All manuals describing the core constructs, codebooks, elements of the instrument, and the instrument itself are available online (http://www.show.wisc.edu).

**Table 1:** **Wisconsin Assessment of Social and Built Environment Domain**

Domain	Description of features	Outcomes assessed	Citations
Neighborhood characteristics	Features related to the sensory experience of the neighborhood including aesthetics, presence of shade trees, presence of publically available amenities such as seating/benches or public art, and presence of neighborhood signs	obesity, physical activity, activity-friendly communities, walking to work, walkability, active commuting to school, active transport, depression	[4, 5, 9, 14, 15, 18, 19, 29–42]
Transportation environment	Features that facilitate safe and efficient movement and active transportation throughout the environment including traffic volume, street type, presence of sidewalks and bike lanes, and presence of public transit	obesity, activity-friendly communities, walking to work, urban bicycling and walking	[14, 28–31, 33, 42–44]
Destinations/Land use	Factors concerning the availability or accessibility of nearby facilities whether residential or non-residential and the diversity of land use	active commuting to school, obesity, active transport, physical activity, mental and physical self-reported quality of life, self-rated health, urban bicycling and walking	[2, 14, 17–19, 28, 32, 33, 39–43, 45–50]
Social Environment	Aspects related to neighborhood social capital and presence of a protective social community including presence of individuals partaking in positive activities, social gathering places, and safety from crime	obesity, physical activity, activity-friendly communities, walkability, active commuting to school, health-related quality of life	[8, 9, 14, 15, 18, 19, 29, 30, 32, 36, 37, 39–41, 44, 51]
Connectivity	Features related to directness of travel routes including intersection density, average block length, and presence of pedestrian cross-walks, sidewalks, and bike lanes	active commuting to school, active transport	[14, 17, 33, 41, 47, 48, 52, 53]

The majority of items on the instrument were posed as dichotomous yes/no or presence/absence of features. Counts, frequencies, or quantities were also included to capture features such as speed limits, numbers of non-residential destinations, and quality of the aesthetics such as presence or absence of litter and graffiti. Two novel features of the instrument are (1) the inclusion of items to analyze road/street intersections, quantifying curb cuts and ramps, crosswalks, pedestrian safety signs and devices, and traffic frequency to assess walkability and connectivity within the neighborhood; and (2) elements to capture social aspects of the environment that may encourage or hinder outdoor physical activities among neighborhood residents, such as the presence of individuals exercising, engaging in hostile activities, etc.

### Defining neighborhood level exposures

**Figure 1:**
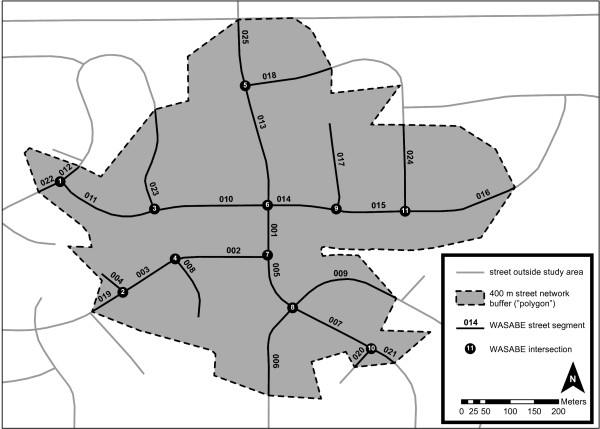
**Example of a 400-meter non-Euclidian street-network buffer for measuring neighborhood environment around select- participant household.**

In order to define “neighborhood” environments, ArcGIS Network Analyst (ESRI, Redwood, CA) was used to define a 400-meter (about a quarter of a mile) non-Euclidian street network buffer around each selected household. This distance (equivalent of a 5–10 minute walk) was chosen because previous studies on “walkability” have found it to be the upper limit of the distance individuals are generally willing to walk to procure a service [28, 48, 54]. The resulting street network polygon includes a representation of the routes pedestrians and cyclists normally rely on for travel around each household (see Figure 1) [48, 54]. Within polygons, units of analyses were defined as street segments and intersections. The distance between two intersections, or from one intersection to the edge of the polygon boundary, was termed a segment. Segment lengths were set at a maximum of 400 meters (common in more rural areas) and minimum of 6 meters. Intersections were defined as a point from which an observer, pedestrian, or driver has to choose between two or more different directions to continue walking and/or driving (excluding driveways).

### Data collection, training and field operations

The research team developed a manual of operations with detailed instructions for the implementation of the WASABE instrument. Undergraduate and graduate students were recruited as field surveyors. All field surveyors participated in an intensive three-day training session on protocol and data collection methods and up to two weeks of field practice prior to any field data collection. Field surveyors were assigned specific polygons surrounding households included in the SHOW sample and provided with corresponding maps that included enumerated segments and intersections to be measured within every polygon. The time to complete data collection for all segments and intersections within a polygon varied greatly depending on polygon characteristics, such as total number of segments, segment lengths, or presence or absence of features, with an average range between 4–8 minutes.

Data were gathered in the summers of 2010 and 2011 for a select number of 2009 (n = 65) and 2010 (n = 618) SHOW participant households. Participant selection in 2009 was a convenience sample based on proximity to SHOW headquarters as well as ability for gathering data across all levels of urbanicity (urban, suburban, and rural). The convenience sample in 2009 was meant to support instrument development and to refine methods for implementing the survey; therefore, these data were not included in testing of the tool’s construct validity. Lessons learned from the 2009 sample data collection were used to collect more rigorous data in 2011 for the 2010 participants. The 2010 participant sample was the full state-wide representative sample with 15 (1.6%) participants or 10 out of 618 missing at random across the entire state. Summer months were chosen (June to August) to ensure comparability across communities and reduce measurement bias introduced by seasonality. Quality control checks were employed including systematic review of incoming instruments for missing data, incomplete, or illogical responses. It was not economically feasible to have field surveyors return to sites to re-rate them; however, all segments were rated twice during the first two weeks of data collection by different raters to ensure standardization of data collection and identify any field issues early on in data collection. Discrepancies were discussed with field surveyors and used to provide corrective training. Segments were also rated twice in areas where household-specific polygons overlapped and assigned to different raters. Because double rating of segments occurred either on the same day or within one week of each other, these double ratings of segments (N = 882) served as the basis for inter-rater reliability testing. Thus, even if we did not measure intra-rater reliability using standard procedures, our methods provide for some measure of consistency when applied at two different time points. Further, the team spent substantial time looking for repeated patterns of error by rater and consequently cleaning and/or dropping data if inconsistencies were found. This QC process led to any necessary repeat rater trainings.

### Statistical analyses – reliability testing and descriptive analyses

Once data were collected, a “segment level” file was processed and cleaned in order to further assess missing data and calculate item specific inter-rater reliability. From this clean dataset, a second “household level” (i.e., polygon) file was developed to include derived variables such as the presence or absence, counts, and density of selected features within a polygon. For example, for relatively rare items such as parks, a dichotomous variable was created, indicating presence or absence of a park within a household polygon; and for sidewalks, a density measure of sidewalks/total segment length (in meters) in the polygon was derived. Additional prevalence estimates were calculated by dividing the number of segments with a certain characteristic (e.g., the number of segments containing grocery stores) by the total number of segments within the polygon around the individual’s household. Segment and polygon level datasets and codebooks including definitions for all items were created. All statistical analyses were conducted using SAS 9.3 (SAS Institute, Inc., Cary, NC).

### Inter-rater reliability

To better understand the reliability of the tool across different field surveyors, we explored inter-rater reliability using percent agreement across all segments in the dataset that were double rated by different raters within at most one week of one another. We used percent agreement to assess inter-rater reliability rather than kappa statistics because our goals were to assess comparability and reproducibility across a number of different pairs of raters [12, 55, 56]. Categories of inter-rater reliability were predefined as excellent (>90%), very good (80-89%), good (70-79%), moderate (60-70%) or poor (<59%). After initially testing the reliability and validity of the results 2009 data, measures with moderate to poor agreement were dropped from the final WASABE tool used for the 2010 sample collection. In addition, for more subjective measures where we saw poor reliability we improved the training and modified the manual.

### Descriptive analyses - prevalence of selected built and social environmental features

In order to assess construct validity of WASABE, we examined the ability of the instrument to capture variation in exposure to built and social environment features within the SHOW sample. The prevalence of features was examined by sociodemographics, health behaviors, neighborhood perception and census block group urbanicity (urban vs. rural) for 939 participants. We hypothesized that features would vary across socio-economic strata, by neighborhood perceptions and census block group (CBG) levels of economic-hardship and urbanicity.

Urban and rural communities were classified at the census block group level according to U.S. Census definitions for urbanicity (http://www.census.gov/geo/reference/ua/urban-rural-2010.html). Urban was defined by combining “urbanized areas” of 50,000 or more people and “urban clusters” of at least 2,500 and less than 50,000 people; all other areas were defined as rural. These units were chosen in order to distinguish and classify rural “towns” that have more grid-like street-networks from more isolated rural landscapes.

#### Built and social environment data

Dichotomous measures of presence or absence of a feature within the 400 m buffer surrounding an individuals’ household (or within a polygon) were used to estimate prevalence of non-residential destinations, walking/biking trails, parks, fitness centers, grocery stores, litter and trash, and fast food restaurants. Social environment features including presence or absence of neighborhood social or cultural signs, security warnings or signs and active engagement defined by observation of people walking or biking were also derived. Chi-square tests of equal prevalence by different classes of predictor variables were used. A density measure was calculated as the total length of segments in a ploygon with presence of features relative to the total segment length. We used this to explore sample variation by sidewalk density. Linear regression was used to test for significant differences in mean sidewalk density.

#### Sociodemographic factors

Sociodemographic characteristics examined included gender (male vs. female), age group (21–29, 30–39, 40–49, 50–64, ≥65 years), race and ethnicity (non-Hispanic white vs. non-white), marital status (married or living with a partner, never married, divorced or single), education (high school degree or less, some college or college graduate), and levels of family poverty (<100% of the federal poverty level [FPL], 100-199%, 200-399%, ≥400% of FPL).

#### Health behaviors

Health promoting behaviors were classified as dichotomous outcomes according to whether or not individuals self-reported having met U.S. physical activity or dietary requirements (yes vs. no). Physical activity requirements were met if a participant reported 600 Metabolic Equivalent of Task (MET)-minutes/week of moderate or vigorous activity (or the equivalent of 150 minutes of moderate to vigorous activity) [57] and diet requirements met if participants reported consumption of more than 4–5 servings of fruits or vegetables per day [58].

#### Perceived environment

Self-reported agreement (strongly agree or agree vs. disagree or strongly disagree) that neighborhood “is well maintained”, “there are many interesting things to look at”, and “there is easy access to fresh fruits and vegetables in my community” were used to create dichotomous measures of neighborhood perceptions.

#### Economic deprivation

Census block group level socio-economic status (SES) was measured using an economic hardship index (EHI). EHI is a composite index of five measures derived using US Census 2000 data including crowded housing (percentage of occupied housing with more than one person per room); poverty status (percent of persons living below 100% of the federal poverty level); employment (percent of persons over the age of 16 years who are unemployed); education (percent of persons over the age of 25 without a high school education); dependency (percent of the population age under 18 or over 64 years of age); and individual annual income categories (<$20,000; $20,000-44,999; ≥$45,000) [59, 60]. CBGs were ranked based on these indicators and assigned a tertile of economic hardship (low, medium or high).

Because of the two-stage sampling approach used in SHOW [25], SAS survey procedures incorporating sampling design elements and weights were employed to account for the correlation structure (non-independent observations) due to in-home and community clustering, using SAS version 9.3 (SAS Institute, Inc., Cary, NC).

## Results

The results of our literature review (Table 1) demonstrate a growing body of evidence supporting the notion that many aspects of the built and social environment can impact health behaviors and outcomes. Exercise facilities [2, 49, 61, 62], enjoyable scenery [35, 63–66], frequency of seeing others exercise [5, 35, 63–65, 67], presence of and satisfaction with recreation facilities [14, 68, 69], presence of nonresidential destinations [18, 29, 30], sidewalks [2, 28, 30, 63, 70], and lower levels of physical disorder [6] have been linked to higher levels of physical activity. Other features including higher rates of crime and proximity to fast food restaurants have been associated with increased body mass index (BMI) in adults and children [5, 6, 34, 71, 72]. Mental health and self-rated health have also been found to correlate with neighborhood built and social environments [7, 8, 51, 70].

### Inter-rater reliability

Consistent with other audit based tools, inter-rater reliability for the majority of items within WASABE was high with an overall range of percent agreement (PA) between 54% and 100%. Of the 115 derived items assessed, 81 (70%) had excellent PA above 90% with the majority of items (81%) with a percent agreement above 95%. Approximately 14 items (12%) had very good agreement between 80-89% and 20 items (17%) had good or moderate agreement less than 80%. Table 2 presents results of inter-rater reliability for questions grouped according to a select number of items corresponding to features identified within each of the pre-specified domains. A more detailed description of all items percent agreement is available in additional files (see Additional file 2: Table S5). The domain with the greatest proportion of items with only good to moderate agreement compared to very good and excellent was neighborhood characteristics. Items pertaining to both positive and negative neighborhood aesthetics had moderate to poor PA (e.g., “Does the street segment have […]?” neglected vegetation [PA = 71%] or careless and harmless litter [PA = 53%]). In contrast, items pertaining to negative advertisement and presence of graffiti as well as public amenities such as trash cans, benches, bike racks, and public art of buildings present in the segment (residential, non-residential, and recreation facilities) had the highest percent agreement (all PA >95%).

**Table 2:** **Percent agreement of select items by domain**

Domain	Features	Items	% Agree	95% CI
**Neighborhood characteristics**	Positive aesthetics	Variation in building materials and colors	66.7	65.3 - 68.2
		Vegetation	70.5	69.0 - 71.9
	Negative aesthetics	Buildings in poor condition	76.4	75.1 – 77.8
		Vegetation neglected	70.8	69.3 – 72.2
		Careless/harmless litter	53.5	52.0 – 55.1
		Broken/boarded up windows	98.0	97.6 – 98.5
		Fast food advertisements	99.8	99.7 – 99.9
	Advertisements	Alcohol advertisements	98.1	97.6 – 98.5
	Public amenities	Public trash cans	95.7	95.1 – 96.3
		Seating/benches	94.7	94.0 – 95.4
		Bike racks	97.1	96.6 – 97.6
		Public art	98.5	98.1 – 98.9
		Public attractive natural features	95.1	94.4 – 95.7
**Transportation environment**		Sidewalks	80.9	79.7 – 82.1
	Transportation	Speed limit	81.9	77.7 – 86.1
		Public transportation	87.5	86.4 – 88.5
		Pedestrian safety signs (segment)	90.6	89.7 – 91.5
		On-street parking with bulb-out (segment)	99.1	98.8 – 99.4
		On-street parking without bulb-out (segment)	87.4	86.3 – 88.4
		Buffer between street and sidewalk	93.1	92.2 – 94.0
		Major misalignments/cracks in sidewalk	85.2	83.9 – 86.5
		Number of traffic lanes (segment)	89.8	88.9 – 90.8
**Destinations and land use**		Street type	91.4	90.5 – 92.3
	Land use diversity	Single family homes	85.7	84.6 – 86.8
		Multi–unit homes (2–6 units)	79.5	78.2 – 80.7
		Apartment building/complex (>6 units)	92.4	91.6 – 93.3
		Mobile home or trailer park/community	100.0	100.0 – 100.0
		Farm complexes	100.0	0.99 – 100.0
		Off-road walking/biking trails or paths	95.3	94.6 – 95.9
		Undeveloped land/farmlands/woodlands	95.2	94.6 – 95.9
		Number of stories of tallest building in segment	72.6	71.1 – 74.0
		Type of building (tallest building)	85.1	84.0 – 86.2
		Topography	81.0	79.8 – 82.2
		Abandoned buildings	99.3	99.0 – 99.6
**Non-residential destinations**	Educational	Schools	97.7	97.2 – 98.2
Assets	Recreational	Parks or designated green spaces	93.5	92.7 – 94.3
		Indoor fitness facilities	99.9	99.8 – 100.0
		Sports/playing fields, courts, or tracks	96.8	96.3 – 97.4
		Playgrounds or splash pads	96.5	95.9 – 97.1
		Pools (indoor or outdoor)	100.0	100 – 100.0
	Restaurants	Other restaurants	97.2	96.7 – 97.8
		Coffee shops	99.6	99.4 – 99.8
	Food outlets	Food supermarkets or grocery stores	99.9	99.8 – 1.00
		Convenience stores or gas station stores	99.8	99.6 – 99.9
		Gas stations	99.7	99.6 – 99.9
		Pharmacies	99.4	99.2 – 99.6
	Health Care	Health care facilities	98.7	98.4 – 99.1
		Retail stores	97.2	96.7 – 97.8
	Retail	Indoor malls, department stores, or “big box” stores	100.0	100.0 – 100.0
		Service providers	93.2	92.4 – 94.0
	Fitness	Indoor fitness facilities	99.9	99.8 – 100.0
		Cultural entertainment facilities	99.1	98.8 – 99.4
		Non-religious community centers	99.4	99.2 – 99.6
	Religious	Church, synagogue, mosque, or other religious centers	98.0	97.6 – 98.5
	Office and work space	Office space	98.3	97.9 – 98.7
		Warehouses	98.3	97.9 – 98.7
Detriments	Alcohol and liquor outlets	Bars/night clubs	99.2	98.9 – 99.5
		Liquor/tobacco stores	99.5	99.3 – 99.7
	Fast food	Fast food restaurants	98.9	98.6 – 99.2
**Social environment**	Signs of social capital	Neighborhood social/cultural message or event	79.7	78.5 – 81.0
		Political message or event	81.2	80.0 – 82.4
		Religious message or event	97.2	96.7 – 97.8
		Security warning signs	81.9	80.7 – 83.0
	Active engagement	People walking	67.4	65.9 – 68.9
		People bicycling	75.0	73.6 – 76.3
**Street connectivity**		Pedestrian crosswalks (intersection)	73.3	71.3 – 75.2
		Pedestrian crosswalks worn off (intersection)	91.1	89.8 – 92.3
		Medians/pedestrian islands (intersection)	93.7	92.6 – 94.8

Items that were potentially time-dependent were also found to have moderate to good percent agreement versus very good or excellent PA including observations of the number of people walking (PA=67%) and bicycling in the segment (PA=75%). Within land use measures, building height was the only item with good (PA=72%) compared to very good or excellent PA for all other features. Intersection features also had good percent agreement including crosswalk presence (PA=73%) and excellent agreement for presence of medians and pedestrian islands (PA=94%), which aid in pedestrian safety for crossing the street.

### Prevalence of built and social environmental features

Prevalence of built environment features found in neighborhood environments varied significantly according to individual level socio-demographics, neighborhoods, and community context. Table 3 presents prevalence of non-residential destinations, walking and bicycling trails, sidewalks and parks by individual level socio-demographic strata. Significant variation in presence and density of features across all strata were observed. Individuals 21–29 years of age, non-whites, individuals never married, and lower family income were more likely to live in neighborhoods with non-residential destinations, compared to older age groups, whites, married and individuals with incomes ≥200% of the Federal Poverty level (all p < 0.001). Presence of parks and sidewalk density were also higher in neighborhoods surrounding younger and low-income participants (both p < 0.001). The prevalence of walking and biking trails also varied significantly by age and marital status, with younger individuals (less than 29 years old), and those having never married living in neighborhoods with higher prevalence of walking and biking trails (both p < 0.0015) compared to their respective counterparts. In contrast, presence of fitness centers varied significantly across levels of all community characteristics examined (data not shown see Additional file 2: Tables S6 and S7). More fitness centers were identified in neighborhoods surrounding individuals with greater than a college degree compared to high school or less.

**Table 3:** **Sociodemographic characteristics and prevalence of features surrounding 2010 participant households**

	Total population (n =939)	Total population	Non-residential destinations	Walking and biking trails	Sidewalk density*	Parks
Individual demographics	n	(weighted %, 95% CI)	(row %, 95% CI, Chi Square)	(row %, 95% CI, Chi Square)	(row %, 95% CI, ANOVA)	(row %, 95% CI Chi Square)
**Gender**			0.13	0.87	0.74	0.44
Male	421	50.1 (47.9-52.3)	52.7 (44.1-61.3)	22.2 (13.4-31.1)	43.5 (35.3-51.7)	40.0 (32.2-47.8)
Female	518	49.9 (47.7-52.3)	56.1 (48.7-63.6)	21.8 (15.0-28.7)	44.3 (36.8-51.8)	42.4 (34.9-50.0)
**Age**			**<0.001**	**<0.01**	**<0.001**	**0.03**
21-29	155	19.8 (14.6-24.9)	75.2 (62.2-88.2)	39.6 (18.6-60.6)	66.9 (54.6-79.2)	54.8 (41.6-68.0)
30-39	148	18.0 (14.6-21.5)	52.7 (40.1-65.2)	14.8 (5.9-23.7)	51.1 (40.8-61.4)	41.3 (30.7-51.9)
40-49	204	22.1 (18.5-25.7)	47.1 (37.7-56.6)	20.6 (11.2-30.1)	36.7 (27.2-46.1)	33.8 (24.0-43.6)
50-64	311	29.6 (25.3-33.9)	50.5 (40.0-61.0)	16.6 (10.0-23.1)	32.4 (23.5-41.3)	39.2 (29.0-49.3)
≤ 65	121	10.5 (8.5-12.5)	44.8 (32.0-57.5)	19.9 (10.5-29.2)	29.9 (20.4-39.4)	36.9 (23.7-50.2)
**Race/Ethnicity**			**<0.001**	0.74	**<0.001**	**0.02**
White (Non-Hispanic)	832	87.5 ((84.6-90.3)	51.0 (43.1-59.0)	21.8 (14.0-29.7)	40.5 (32.7-48.3)	39.3 (32.1-46.5)
Non-white	104	12.5 (9.7-15.4)	79.0 (71.2-86.9)	23.5 (12.7-34.3)	67.9 (58.1-77.6)	54.9 (41.0-68.7)
**Marital status**			**<0.001**	**0.01**	**<0.001**	**<0.001**
Married, with partner	611	65.1 (59.4-70.8)	43.2 (34.5-52.0)	18.3 (10.9-25.6)	33.7 (25.6-41.8)	33.9 (25.9-41.8)
Never married	176	21.1 (15.7-26.5)	83.5 (74.0-93.0)	36.8 (17.2-56.4)	73.1 (63.5-82.7)	58.5 (45.4-71.6)
Single (divorced, widowed)	150	13.8 (10.9-16.6)	63.7 (52.4-75.0)	17.7 (9.8-25.5)	47.3 (36.2-58.5)	50.2 (39.0-61.5)
**Education status**			0.30	**0.09**	0.74	0.31
High School or less	236	24.7 (21.2-28.2)	54.1 (42.7-65.4)	16.0 (7.9-24.2)	42.4 (31.4-53.5)	35.2 (24.9-45.4)
Some college	397	43.1 (39.0-47.3)	58.1 (50.3-65.9)	21.2 (11.8-30.6)	45.2 (36.7-53.6)	44.1 (35.8-52.4)
College or beyond	304	32.2 (27.1-37.4)	50.2 (38.8-61.6)	27.9 (17.3-38.5)	43.3 (33.2-53.3)	42.0 (47.4-68.6)
**Family income**			**<0.001**	0.47	**<0.001**	**0.04**
<100% of FPL	105	12.5 (9.0-15.9)	80.0 (67.8-92.1)	25.8 (8.4-43.2)	67.3 (54.5-80.0)	48.2 (33.0-63.4)
100-199% FPL	144	15.5 (12.1-18.8)	69.2 (58.0-80.5)	27.9 (14.2-41.6)	55.0 (43.9-66.0)	51.7 (39.4-63.9)
200-399% FPL	327	33.5 (29.6-37.5)	51.5 (42.3-60.8)	20.5 (12.3-28.6)	37.9 (29.5-46.3)	40.4 (31.8-49.0)
400% + FPL	314	33.1 (28.9-37.4)	40.0 (30.6-49.5)	21.2 (11.9-30.6)	34.1 (24.8-43.6)	33.7 (24.9-42.5)
Unknown	49	5.4 (3.8-7.1)	59.4 (43.4-75.4)	11.4 (1.1-21.8)	55.3 (41.0-69.5)	45.9 (29.6-62.2)

*Sidewalk Density is a measure of total sidewalk length per total segment length within a buffer. All tests for statistical significance are bolded when p<.05.

Prevalence of non-residential destinations was higher in neighborhoods with individuals meeting recommended guidelines for physical activity (73%) vs. those that did not (64%) (Table 4). There was no significant difference in prevalence of any features examined in neighborhoods of individuals according to their reported fruit and vegetable consumption.

**Table 4:** **Prevalence of features surrounding 2010 participant households by strata of health behaviors, neighborhood perceptions, and census block group economic hardship and urbanicity**

	Total population (n =939)		Non-residential destinations	Walking and biking trails	Sidewalk density*	Parks
Health promoting behaviors	n	(weighted %, 95% CI)	(row %, 95% CI, Chi Square)	(row %, 95% CI, Chi Square)	(row %, 95% CI, ANOVA)	(row %, 95% CI Chi Square)
**Meet physical activity recommendations (>600 MET/MIN/WEEK)**			0.22	0.86	0.75	0.48
Yes	716	76.6 (73.2-80.0)	53.4 (45.3-61.5)	22.2 (14.2-30.1)	43.6 (35.5-51.7)	40.5 (32.9-48.0)
No	223	23.4 (20.0-26.8)	57.9 (48.9-67.0)	21.6 (13.5-29.7)	44.9 (36.0-53.9)	43.7 (34.3-53.1)
**Servings of fruits and vegetables (4–5 daily)**			0.15	0.75	0.18	0.27
Yes	155	17.1 (13.8-20.4)	48.2 (36.1-60.2)	22.8 (12.3-33.3)	37.1 (25.9-48.4)	44.6 (32.2-56.9)
No	683	82.9 (79.6-86.2)	55.7 (47.4-63.9)	21.4 (13.0-29.8)	44.0 (35.9-52.1)	38.8 (31.7-45.9)
**Neighborhood perceptions**						
**Many destinations within easy walking distance**			**<0.001**	**<0.001**	**<0.001**	**<0.001**
Agree	445	58.7 (50.3-67.1)	69.6 (61.3-77.8)	30.6 (19.4-41.8)	59.5 (51.0-68.1)	53.6 (45.3-61.8)
Disagree	391	41.3 (32.9-49.7)	32.5 (24.8-40.3)	8.7 (4.3-13.1)	19.2 (12.0-26.5)	20.1 (13.7-26.6)
**Many interesting things to look at**			0.89	**0.0010**	**0.03**	0.11
Agree	645	78.6 (75.0-82.3)	54.1 (45.2-62.9)	24.4 (14.8-34.0)	45.0 (36.7-53.3)	41.5 (33.6-49.3)
Disagree	190	21.4 ((17.7-25.0)	54.8 (44.2-65.3)	10.6 (4.8-16.3)	35.0 (25.644.3)	33.8 (24.4-43.1)
**Community well maintained**			**0.01**	0.63	**0.01**	0.30
Agree	747	89.6 (87.3-92.0)	52.4 (43.8-60.9)	22.0 (13.7-30.3)	41.4 (33.4-49.4)	39.2 (31.7-46.7)
Disagree	84	10.4 (8.0-12.7)	70.2 (58.5-81.8)	18.9 (4.8-33.0)	55.9 (44.1-67.7)	45.1 (33.5-56.8)
**Easy access to fresh fruits and vegetables**			0.24	0.47	0.92	0.86
Agree	713	86.6 (83.2-90.0)	53.2 (44.5-62.0)	22.0 (13.4-30.5)	43.0 (34.7-51.2)	39.6 (31.9-47.3)
Disagree	123	13.4 (10.0-16.8)	61.0 (48.7-73.3)	18.8 (9.1-28.5)	42.5 (32.1-52.8)	40.6 (29.6-51.5)
**Census block group characteristics**						
**Economic hardship**			**0.02**	0.83	**0.03**	0.19
Low	362	39.0 (28.2-49.9)	50.3 (37.2-63.4)	24.3 (11.4-37.2)	43.6 (29.3-57.9)	40.4 (28.6-52.1)
Medium	312	33.1 (21.2-44.9)	43.7 (28.2-59.2)	18.8 (8.3-29.2)	30.2 (15.4-45.1)	33.1 (19.3-47.0)
High	265	27.9 (19.5-36.2)	73.0 (60.1-85.8)	22.8 (6.0-39.6)	60.6 (47.8-73.4)	52.0 (37.1-66.9)
**Urbanicity (Census 2010 urbanized areas & urban clusters)**			**<0.001**	**<0.001**	**<0.001**	**<0.001**
Urban	587	69.2 (60.2-78.1)	67.9 (58.8-77.1)	28.6 (19.0-38.7)	61.6 (53.0-70.3)	54.0 (46.3-61.7)
Rural	352	30.8 (21.9-39.8)	24.2 (14.7-33.6)	6.8 (1.9-11.7)	4.2(0.6-7.7)	12.5 (2.2-22.8)

*Sidewalk Density is a measure of total sidewalk length per total segment length within a buffer. All tests for statistical significance are bolded when p<.05.

When examining differences in neighborhoods classified based on individuals’ perceptions, prevalence of non-residential destinations was 75% in neighborhoods for those who agreed that there were many destinations within walking distance compared to 54% in those who disagreed. Prevalence of walking and biking trails, parks, and sidewalk density were also significantly higher in neighborhoods of individuals who strongly agreed that there were many destinations compared to those who disagreed (p < 0.0001 for all comparisons of agreement and feature). There was also a higher prevalence of walking and bicycling trails and sidewalk density among those who agreed that there were many interesting things to look at in their neighborhood compared to those who disagreed (p = 0.001 and p < 0.03). Prevalence of non-residential destinations and sidewalk density were statistically lower among those who agreed that their neighborhoods were well-maintained vs. those that disagreed (p = 0.006 and p = 0.008, respectively). No significant variation in prevalence or density were observed based on individuals perceptions that fruit and vegetables were easily accessible in a neighborhood.

Trends in census block group SES were similar to individual categories of SES, higher prevalence of non-residential destinations and sidewalk density were observed in residents of lower SES/high EHI census block groups. Prevalence of most features previously used to describe “walkable” or “active living communities” (e.g., sidewalks) were found more often in urban compared to rural communities.

Distribution of sociodemographics, neighborhood perceptions and census block level socio-economic status by social environmental features such as neighborhood social or cultural messages, security warnings or signs were also found (data not shown). Similar trends in variation of features were observed with younger age groups living in neighborhoods with more neighborhood or social messages and active engagement such as walking or biking compared to younger ages for example. Prevalence of security warnings or signs was greater in neighborhoods of non-white vs. white (p < .0001) and never married compared to married or divorced or widowed individuals (p < .0001). Social and cultural messages and active community engagement were also more prevalent in neighborhoods surrounding individuals who agreed there were many destinations and interesting things too look at compared with individuals who disagreed.

## Discussion

The WASABE instrument has proven overall to be a reliable and valid audit-based tool for examining the effects of the social and built environment on health and health promotion. Overall inter-rater reliability was high with average percent agreement within each domain close to 90%. The majority of items were based on previously developed items with very good to excellent percent agreement, and the application and use within the WASABE tool confirmed their reliability. Moderate to poor PA was most often associated with features that can be difficult to observe from the street such as housing type (single vs. multi-family units), or that are more subjective in nature (e.g., major misalignments or cracks in sidewalks).

We also found the instrument has good construct validity, as most significant differences in presence or absence of features were found in the direction that one might expect; for example, neighborhood destinations and sidewalk density were greater in urban and small urban clusters. Furthermore, there is growing evidence to suggest younger age groups are choosing to live in more walkable urban areas, and our data suggest this is also true with greater than 75% of participants less age 21–29 years old were living in areas with neighborhood destinations compared to 45% for individuals over the age of 65. At the same time, sidewalk density and neighborhood destinations were also significantly higher in neighborhoods surrounding participants with combined family incomes less than 100% of the federal poverty level, likely these participants are living in more socially isolated urban areas underscoring the complexity of relationships between social and built environments and health. Variation by socio-demographic, neighborhood, and community context is reflective of the diverse features of the physical landscape and different land use patterns in the state of Wisconsin. Wisconsin offers a unique landscape and study area to explore how features of the built environment predict health and health behaviors because of this diversity in both features and across SES strata. These findings are also consistent with emerging research which suggests that one mode of data collection on built and social environment features is not sufficient and a combination of approaches may provide the best measurement [20].

In combination, the SHOW and WASABE data provide important resources for neighborhood and community level social and built environment assessments. Mounting evidence shows that neighborhood and individual level socio-economic position are independently associated with adverse cardiovascular and other metabolic outcomes, but mechanisms by which these factors affect behaviors and physiologic outcomes are still largely unknown [11, 30, 73]. Objective audit tools such as WASABE are needed to refine measures used to move the field forward in understanding of the complex pathways by which neighborhood environments affect overall health status as well as chronic disease and health promoting behaviors such as physical activity across the life-course [4, 5, 11, 27, 73–75].

WASABE builds on previous research exploring the use of systematic social observation and objective audits to provide an unbiased, population-level measure of community social and built environments [9, 14–16, 18, 19]. Few studies have examined the association between the built and social environment, and health outcomes using a probability-based sample and/or in rural communities. On average data collection per segment was 4–8 minutes, but this range varied depending on whether or not raters were in an urban vs. rural community. Many rural communities did not have as many features to inventory, thus the mean time for data collection in these communities was shorter than in urban communities where the average was higher in the range of 8–11 minutes. To our knowledge, no research has studied these questions in the context of a statewide health survey gathering the breadth and depth of information being gathered by SHOW.

Results from this study also suggest the WASABE audit tool is robust and can be employed in a variety of settings. It has been shown to be discriminatory across socio-economic strata as well as diverse levels of urbanicity [14, 71, 76]. This is particularly important for studies in rural communities where assessments of built environments are often overlooked [41, 65]. The majority of features identified within the WASABE tool were found to differ in prevalence or density in urban compared to rural communities. Urbanicity was defined according to U.S. census definitions and included large urbanized areas and small urban clusters found throughout rural Wisconsin. The commonality of the two is that land use in small town centers are built on a grid network similar to a more urbanized area but on a much smaller scale. We combined these small urban cluster neighborhoods with larger metro communities from remote rural areas and found significant differences in prevalence of built environment features known to promote active living [14, 71, 76]. The majority of items including access to trails and recreational facilities, parks, intersections, sidewalks, signage and aesthetics and measures of social engagement are all relevant items to consider in rural communities, particularly in smaller rural towns and town centers. When a resident lives on a country road, with very low traffic, items like crosswalks, traffic lights, and other signage may not apply. However, availability of walking/biking paths, traffic volume, and aesthetics as well as perception of open recreational areas are all still important and relevant items to consider. In the future, results from this project will offer an important opportunity to further explore and refine measurement in rural communities.

More work is needed to discern how these features are related to associations in other measures of health and quality of life and to discern barriers to healthy living in more rural communities [10, 2, 65]. The tool provides systematic methods to measure features of the environment at the same time offering the flexibility to measure features across different land-use and community environments and contexts. For the purpose of initial data collection a 400-meter buffer was drawn and rated to represent a person’s community with the centroid of that community being the individual’s household. However, given that rating occurs on a per segment level, a different size buffer could be drawn (i.e., 800 meters) or a different centroid of analysis (such as a school or place of work) could be used to define units of observation. The tool may also be useful for assessing children’s environments. Some of the domains captured by the instrument, such as access to recreational facilities, including parks and commercial facilities, have been found to be associated with children and adolescents’ physical activity [77]. Sidewalks and bike lanes increase the likelihood that children will walk or bike to school [77]. Other domains captured by WASABE have recently been shown to increase the effect of family based obesity interventions for children [78].

One aspect to note is that we deliberately conducted data collection with this instrument during summer months to ensure comparability across communities that may be more limited during other winter months when seasonal issues such as snowfall may distort measurement of important environmental features. Use of this tool in other regions with similar seasonal variations in weather should consider how measurement of features and items may be affected. To that end, the tool includes an element for tracking weather that can be used in the analysis to account for weather variability if needed.

Measuring neighborhood effects on health requires deliberate evaluation and assessment--not all measures will be relevant for every health question. In order to truly understand the interaction between neighborhood environments on health, flexible tools that explore a number of features and domains simultaneously are needed. Each item in the WASABE scale is independent, meaning items can be dropped if not relevant for the study context. Given the flexibility in design, this study instrument could be applied in other settings, such as in dense European urban centers where likely different features would be prominent in the analyses.

Our WASABE data can also be analyzed for comparison of both individuals’ perceptions and readily available objective data, using the SHOW neighborhood perception questionnaire, as well as both public and commercially available extant GIS data, respectively. This will allow both for triangulation of multiple types of measurements, as well as instrument refinement by examining the predictive value of the objective audit compared to GIS, and the relative merits of each method [9, 10, 20]. These methods will be important for disentangling the complex relationships between social, built, and socioeconomic environments on health disparities for current as well as future generations [5, 72, 79]. Also, these tools will be important for better understanding the complex role that neighborhoods have in contributing to persistent issues of health equity both in and outside the United States [2, 4, 6, 7, 34].

Another unique aspect of this audit tool is the careful assessment of street intersections. To our knowledge, while several tools previously used in the US [15], Australia [19] and New Zealand [11] did include intersections as an element, none of these audit tools have examined intersection features to the level of scrutiny of the WASABE instrument in a population-based sample. In an attempt to better capture some components of connectivity, such as connectedness of pedestrian crosswalks and walk/don’t walk signals; this intersection analysis provides new information for researchers interested in gauging the affects of the intersection characteristics on walkability. Though not all of these features had exceptionally high inter-rater reliability, possibly due to rater fatigue, this intersection assessment provides a higher resolution inspection of potential barriers or supports for pedestrians that allow them to move with ease throughout their neighborhoods [15, 17, 19, 27].

There are additional features besides the intersectional analysis that distinguish the WASABE instrument from the other tools which preceded it and guided its development (Systematic Pedestrian and Cycling Environmental Scan, the Walking Suitability Assessment Form, the Analytic Audit Tool, the St. Louis Active Neighborhood Checklist, Irvine-Minnesota Inventory, and the Pedestrian Environment Scan). The first distinguishable feature of the WASABE is its inclusion of elements reflecting social aspects of the environment. The WASABE required surveyors to count the number of people engaging in positive activities or behaviors relevant to building social capital in the neighborhood or conducting physical activity (e.g., running or bicycling) and the number of people exhibiting threatening or hostile behaviors. Though these observations will differ based on time of audit, this is the first attempt at capturing features of the social environment that may influence residents’ likelihood of engaging in physical activities, such as walking, jogging, biking, outdoors. These features cannot be assessed by GIS. Secondly, weather during the time of the scan is also not usually recorded; whereas, the WASABE surveyors took note of the weather during the audit, which can be then considered when examining the traffic count and number of role models in the buffer. Lastly, topography of the segments, traffic volume, and architectural variety are other features, which did not consistently exist in all of the model audit tools, but were included in the WASABE.

Despite overall strengths of the WASABE tool, a few important limitations remain. One particular aspect is a limited understanding of the intra-rater reliability of the tool. While it was not feasible to address this issue in the current study, we standardized measurements, developed a rigorous field data collection manual, focused on consistency of measurement in training and conducted rigorous quality control during field data collection and careful assessment of inter-rater reliability among raters. Despite these efforts, we continued to observe poor inter-rater reliability for features in the neighborhood aesthetics domain. Aesthetics is a very subjective measure; yet, it is a feature of the environment for which may be important in understanding how similar built environments are utilized and can promote health. Our findings are consistent with previous studies that suggest aesthetics are the most difficult to reliably measure using an objective audit [14]. Alternative methods for collecting these data, such as use of community images or ecological momentary assessment that includes a qualitative review, which may provide improved measures of these features relative to those derived by on the ground audits [11, 66]. Finally, further investigations are warranted to improve our understanding of the predictive validity of this tool, relative to other survey or extant GIS based measures.

## Conclusion

The WASABE instrument has proven to be a reliable tool offering a resource for use in population-based health research in order to better understand environment and health interactions. Research generated by this tool will advance our understanding regarding the pathways by which the social and built environment impacts health. The integration of the WASABE audit tool with SHOW perception data provides the opportunity for greater depth of study of the influences of neighborhood characteristics on health. In the future, more studies are needed that combine data on multiple features in order to ensure that the use of the instrument in rural communities is truly capturing the most relevant features for promoting health and wellness, an area of built environment research that has been under-studied relative to urban areas. In addition, the WASABE protocol can and should be adopted to support systematic inventories of neighborhood features using a variety of reference points such as school or work environments. This tool may also be instrumental to document disparities in environmental determinants of health behaviors and outcomes, as well as to assess the impact of interventions targeting the built and social environment in specific communities. Furthermore, WASABE data could be used to foster community empowerment and organized efforts to improve environmental conditions in communities subject to health disparities.

## Electronic supplementary material

Additional file 1: **Wisconsin Assessment of the Social and Built Environment (WASABE) Instrument.** (PDF 170 KB)

Additional file 2: **Additional analyses and components have been included in supplemental materials.** A list of these elements is provided below. Table numbers are sequential based on numbering in the full manuscript. **Table S5.** provides the same results that are presented in table two for all derived variables available within the WASABE. **Tables S6 and S7.** provide results of the descriptive statistics for three social environmental features including sings of neighborhood and cultural events, security and warning signs and visibility of individuals actively engaging in walking and biking. These data provide further illustration of the discriminatory elements of the WASABE tool. (PDF 214 KB)

## Abbreviations

GIS: Geographic Information Systems
UW: University of Wisconsin
SHOW: Survey of the Health of Wisconsin
WASABE: Wisconsin Assessment of the Social and Built Environment
MET-min/wk: Metabolic Equivalent of Task (MET)-minutes/week
SES: Socio-economic status
EHI: Economic hardship index
BMI: Body mass index
PA: Percent agreement.
