# Supplementary material for: The Wisconsin Assessment of the Social and Built Environment (WASABE): a multi-dimensional objective audit instrument for examining neighborhood effects on health
Source: BMC Public Health. 2014 Nov 13;14:1165. doi: 10.1186/1471-2458-14-1165 (PMC4289353; doi:10.1186/1471-2458-14-1165)
Supplement: Supplementary file 1 — Additional file 1: Wisconsin Assessment of the Social and Built Environment (WASABE) Instrument. (PDF 170 KB) [file 12889_2014_7414_MOESM1_ESM.pdf]

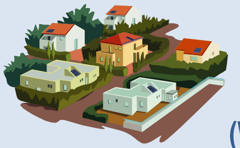

**(WASABE)**
**Wisconsin Assessment of the Social and Built Environment**
Survey of the Health Of Wisconsin

**Date:**

**Day of Week:**

**Start Time:** **Stop Time:**

**Data Collected by** (check all that apply):
__**Foot** __**Auto**

**Assessor ID:**

**Initials:**

**Topography:** ☐ **Mostly Flat** ☐ **Moderately Hilly** ☐ **Mostly Hilly**

**Classification:** ☐ **Urban** ☐ **Suburban** ☐ **Rural**

**Household ID:** **Block Group:**

**Polygon ID:**

**Segment ID:** **Segment length:**
(only if it is a street network terminal)

**Intersection(s) ID:**
(only if it needs to be observed - use page 4)

Weather Conditions:
Temperature (check one):
___very cold (below 20°F)
___cold (20-39°F)
___cool (40-54°F)
___mild (55-69°F)
___warm (70-84°F)
___hot (85-95°F)
___very hot (over 95°F)
Temp. (& wind speed if applicable) check one:
___were based on an official source
___were estimated by observer
Weather Description (check all that apply):
___sunny to partly cloudy
___mostly cloudy to cloudy
___light to moderate rain
___heavy rain or thunderstorm
___light to moderate snow or freezing rain
___moderate to heavy snow, freezing rain
___heavy fog
___snow on the ground
___ice on the ground
___strong winds (approx. 25 mph or higher)

**1.** What **types** of residential buildings are present in the segment?

| **Residential Buildings** | **Yes** | **No** |
|---|---|---|
| a. Single family homes | | |
| b. Multi-unit homes (2-6 units) | | |
| c. Apartment building/complex or condominium (>6 units) | | |
| d. Apartment over retail in multi-story building | | |
| e. Mobile home or trailer (do not count if part of a trailer park) | | |
| f. Mobile home or trailer park/community | | |
| g. Other (Specify): | | |

**2.** How many of each type of non-residential building are present in the segment?
If present, how many are adjacent to the sidewalk (# Adj to SW)?

| **Non-Residential Businesses (1/3)** | **#** | **# Adj to SW** |
|---|---|---|
| a. Bars/nightclubs | | |
| b. Liquor/tobacco stores | | |
| c. Wine stores | | |
| d. Fast food restaurants (i.e., you order and you bring your food to your table) | | |
| e. Other restaurants (i.e., sit-down restaurants; you order & wait staff brings your food to table) | | |
| f. Coffee Shops (e.g., Ancora, Alterra, or local) | | |
| g. Specialty/Ethnic Food Store | | |
| h. Food supermarkets or grocery stores | | |

| **Non-Residential Buildings or Businesses (2/3)** | **#** | **# Adj to SW** |
|---|---|---|
| i. Convenience stores or gas station stores | | |
| j. Gas Station | | |
| k. Pharmacies, drug stores (primarily non-food; e.g. Walgreens, locally owned pharmacies, etc.) | | |
| l. Health care facilities (e.g., hospital, elderly residence, clinic, dentist, acupuncturist, etc.) | | |
| m. Retail stores (e.g., video rental, florist, bookstore, clothing, sporting goods, etc.) | | |
| n. Indoor malls, super centers, department stores, “big box” stores (e.g., Walmart, Home Depot, etc.) | | |
| o. Service providers (e.g., hair salon, bank, laundromat, dry cleaners, accountant, car wash, car repair, post office, FedEx, etc.) | | |
| p. Indoor Fitness facilities (e.g., gyms, fitness centers, health clubs, yoga, martial arts, or dance studios, etc. | | |
| q. Cultural entertainment (e.g., movie theaters, museum, library, etc.) | | |
| r. Other entertainment (e.g., bowling alley, karaoke, billiards, etc.) | | |
| s. Non-religious community center (e.g., senior center, neighborhood center, drop-in center, etc.) | | |
| t. Church, synagogue, mosque, other religious center | | |
| u. Educational facilities (e.g., university, tech school, K-12 schools, daycare, ESL facility, etc.) | | |
| v. Government facilities (e.g. police, courthouse, etc.) | | |

| Non-Residential Buildings / Businesses (3/3) | # | # Adj to SW |
|---|---|---|
| w. Transportation facilities (e.g. train or bus station) | | |
| x. Hotels, motels, inns, etc. | | |
| y. Office space | | |
| z. Warehouse, storage facilities, factory, industrial building, etc. | | |
| aa. Farm Complexes (Barn, silo, rural storage facility, etc.) | | |
| bb. Other non-residential (Specify type): ______ | | |

**Addendum: Building size**:

Tallest building in the segment. ☐ **Res.** ☐ **Non-Res.** ☐ **Mixed Use**

How many stories tall is (are) the tallest building(s)? ______

**3.** What types of recreational facilities are visible in the segment?

| Recreational Facilities | Yes | No |
|---|---|---|
| a. Indoor fitness facilities (e.g., gyms, fitness centers, health clubs, yoga, martial arts, or dance studios, etc.) | | |
| b. Sports/playing field, courts, or track | | |
| c. Playground or splash pad | | |
| d. Golf course | | |
| e. Pool (indoor or outdoor) | | |
| f. Off-road walking/biking trail or path | | |
| i. On one side of the road | | |
| ii. On both sides of the road | | |
| iii. Crossing the road | | |
| g. Other Recreational Facilities (Specify Type): ______ | | |

**4.** What is the predominant land use? Check one or two that apply.

| Predominant Land Use | Check 1 or 2 |
|---|---|
| a. Residential buildings/yards | |
| b. Commercial buildings / businesses | |
| c. Public government buildings | |
| d. Manufacturing | |
| e. School/school yards | |
| f. Parking lots or garages | |
| g. Park or designated green space | |
| h. Abandoned buildings | |
| i. Undeveloped land/farmlands/woodlands | |
| j. Other (Specify): ______ | |

**5.** Evaluate the traffic volume and type.

| Traffic Volume |
|---|
| a. What is the speed limit in the segment?<br>**Enter MPH:** ______ check if unknown: ☐ |
| b. Scan the entire segment and count any cars, trucks, and other motorized vehicles driving within the segment during 2 minutes. Check the appropriate box for number of vehicles.<br>☐ **0** ☐ **1-4** ☐ **5-15** ☐ **16-34** ☐ **35+**<br>check if NA: ☐ If NA, give reason: ______ |
| c. Bus or other public transportation present<br>**Yes** ☐ **No** ☐ |

**6.** On what type of street is the segment? Check **ONE** best description.

| Check 1 | Street Type |
|---|---|
| ☐ | Pedestrian only / Closed to motorized traffic |
| ☐ | Pedestrian with limited access to certain motorized vehicles (e.g., buses, delivery trucks, etc.) |
| ☐ | Dead end street (either one way or two way) |
| ☐ | One way street |
| ☐ | Two way street |
| ☐ | Other (Specify): ______ |

**7.** Describe the following **STREET** characteristics **in the segment ONLY** (**not including intersections**)

| Street Characteristics | Enter # | |
|---|---|---|
| a. Traffic lanes (within the segment) | | |
| b. How many pedestrian crosswalks are present? (within the segment) | | |
| i. How many pedestrian crosswalks have missing pieces or sections worn off? (within the segment) | | |
| c. Walk/Don't Walk signals (within the segment) | | |
| d. Ramps or Curb Cuts (within the segment) | | |
| | **Yes** | **No** |
| e. Pedestrian safety signs and devices (within the segment) | | |
| f. Medians or pedestrian islands (within the segment) | | |
| i. Pedestrian Pushbutton on Median | | |
| g. Traffic calming devices (within the segment) | | |
| h. On-street parking available (within the segment) | | |
| i. With Bulb-Out | | |
| ii. Without Bulb-Out | | |
| i. Railroad (within the segment) | | |

**8.** Please answer the following questions regarding **bicycling** conditions and follow the skip patterns

| Bicycle Transportation/Commuting | | | |
|---|---|---|---|
| **On-Road Biking Conditions** | **Yes, both sides** | **Yes, one side** | **No** |
| a. Is there an on-street, paved, marked bike lane? | | | |
| i. If **Yes [for Q.8(a.)]:** Are there any obstructions in the marked bike lane (e.g., drainage gates, parked cars, etc.)? **Continue on to Q.8(a.)(ii.)** | | | |
| ii. If **Yes [for Q.8(a.)]:** Are there any parts of the bike lanes that are missing or worn off? **If Yes: Skip to Q.9** | | | |
| iii. If **No [for Q.8(a.)]:** Is the outermost lane wide enough (~15 ft.) that it would reasonably fit a motorized vehicle and a cyclist side by side? **If No: Skip to Q.9** | | | |
| iv. If **Yes [for Q.8(a.)(iii.)]:** Are there obstructions in the outermost part of the lane (e.g., drainage gates, parked cars, etc.)? | | | |

**9.** Are sidewalks present in the segment?
Choose **one** description below that is the best fit.

| Sidewalk Analysis | |
|---|---|
| ☐ | Sidewalk is present in the entire segment |
| ☐ | Sidewalk is present, but missing some parts in the segment |
| ☐ | Sidewalk is completely or mostly missing (**Skip to Q.10**) |

| **Sidewalk Features** | **Yes > 1/2** | **Yes ≤ 1/2** | **No** |
|---|---|---|---|
| a. Grassy or other buffer area between street/curb and sidewalk | | | |
| b. Major misalignments or cracks in the sidewalk | | | |

**10.** Does the street segment have the following characteristics?

| **Neighborhood Characteristics (1/2)** | **Yes > 1/2** | **Yes ≤ 1/2** | **No** | **NA** |
|---|---|---|---|---|
| a. Do the buildings have a variety of building materials and/or a variety of color? | | | | |
| b. Are there buildings in poor condition? | | | | |
| c. Is there vegetation (not including shade trees)? | | | | |
| i. Is the vegetation neglected / unkempt? | | | | |
| | **0** | **1-5** | **6+** | |
| d. Are there medium/large shade trees within the segment? | | | | |
| e. Are there street lights over or near sidewalks or paths? | | | | |

| **Neighborhood Characteristics (2/2)** | **0** | **1-2** | **3+** | NA |
|---|---|---|---|---|
| f. Litter | | | | |
| i. Careless/Harmless | | | | |
| ii. Hazardous | | | | |
| iii. Cigarette Butts* (see manual) | | | | |
| g. Broken/boarded up windows | | | | |

**11.** Are any of the following publicly available amenities present within the segment?

| **Publicly Available Amenities** | **Yes** | **No** |
|---|---|---|
| a. Public trash cans (nonresidential) | | |
| b. Seating/benches | | |
| c. Bike rack | | |
| d. Public art (e.g., murals, sculptures, urban furniture, neighborhood kiosks, public fountain, etc.) | | |
| e. Public attractive natural features (e.g., notable public landscaping, gardens, parks or green spaces, pond) | | |

**12.** Are the following signs visible in the segment?

| **Neighborhood Signs** | **0** | **1-2 sm** | **1+ lg or 3+ sm** |
|---|---|---|---|
| a. Neighborhood/social/cultural message or event | | | |
| b. Political message or event | | | |
| c. Religious message or event | | | |
| d. Fast food ads (e.g., billboards, signs outside restaurants—other than logo/name only, etc.) | | | |
| e. Alcohol ads (e.g., billboards, special offer ads, neon signs, etc.) | | | |
| f. Tobacco ads (e.g., special offer ads, signs outside stores, etc.) | | | |
| g. Security warning sign or message that suggests crime or risk of crime in the area (e.g., abduction flier, neighborhood watch, no trespassing sign, no loitering sign, etc.) | | | |
| h. Graffiti (See Manual) | | | |

**13.** Are people engaging in the following activities visible within the segment?
If yes, specify if more or less than 5 people.

| **Activities** | **Yes 1-5** | **Yes 6+** | **No** |
|---|---|---|---|
| a. Walking | | | |
| b. Biking | | | |
| c. Engaging in other physical activities (e.g., jogging, playing a sport, etc.); Specify activities: ____________ | | | |
| d. People loitering, acting hostile, threatening | | | |
| e. Homeless people or panhandlers | | | |
| f. Aggressive, uncontrolled dogs or stray animals. | | | |

## INTERSECTIONS Analysis

**Conducted independently from segments**

## Intersection # ______ Polygon ______

(connect segments # ______ , ______ , ______ , ______ , ______)

**14.** Describe the following **INTERSECTION** characteristics:

| Intersection Characteristics | Enter # | |
|---|---|---|
| a. Traffic Lanes (including turning lanes) | | |
| b. Pedestrian crosswalks **(If 1+, see scenarios in the manual)** ☐ A ☐ B ☐ C ☐ D ☐ E ☐ F | | |
| i. How many pedestrian crosswalks have missing pieces or sections worn off? (within the segment) | | |
| c. Walk/Don't Walk signals (See manual) | | |
| d. Ramps or curb cuts (See Manual) | | |
| | **Yes** | **No** |
| e. Pedestrian safety signs and devices | | |
| f. Medians or pedestrian islands | | |
| i. Pedestrian Pushbutton on Median | | |
| g. Traffic calming devices | | |
| **Traffic Volume within the Intersection** | | |
| h. How many cars, trucks, and other motorized vehicles travel through the intersection (in any direction) during 2 minutes? ☐ 0 ☐ 1-4 ☐ 5-15 ☐ 16-34 ☐ 35+ check if NA: ☐ If NA, give reason: ______________ | | |

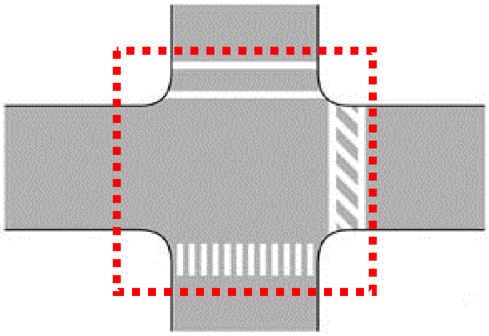

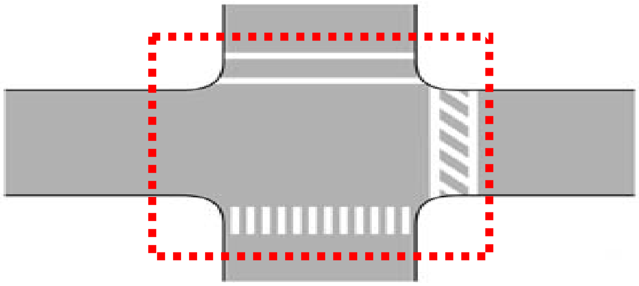

Please use the below space for additional comments: ______________________________
