# Supplementary material for: The Wisconsin Assessment of the Social and Built Environment (WASABE): a multi-dimensional objective audit instrument for examining neighborhood effects on health
Source: BMC Public Health. 2014 Nov 13;14:1165. doi: 10.1186/1471-2458-14-1165 (PMC4289353; doi:10.1186/1471-2458-14-1165)
Supplement: Supplementary file 2 — Additional file 2: Additional analyses and components have been included in supplemental materials. A list of these elements is provided below. Table numbers are sequential based on numbering in the full manuscript. Table S5. provides the same results that are presented in table two for all derived variables available within the WASABE. Tables S6 and S7. provide results of the descriptive statistics for three social environmental features including sings of neighborhood and cultural events, security and warning signs and visibility of individuals actively engaging in walking and biking. These data provide further illustration of the discriminatory elements of the WASABE tool. (PDF 214 KB) [file 12889_2014_7414_MOESM2_ESM.pdf]

Additional File 1

Additional analyses and components have been included in supplemental materials. A list of these elements is provided below. Table numbers are sequential based on numbering in the full manuscript. Table 5. provides the same results that are presented in table two and in addition for all derived variables available within the WASABE. There are over 120 derived variables are included in this full table of inter-rater reliability along with mean estimates of percent agreement by domain. Tables 5 and 6 provide results of the descriptive statistics for three social environmental features including sings of neighborhood and cultural events, security and warning signs and visibility of individuals actively engaging in walking and biking. These data provide further illustration of the discriminatory elements of the WASABE tool.

Table of Contents Additional File 1

Table 5. Percent Agreement of Select Items by Domain (Full)

Table 6. Sociodemographic Characteristics and Prevalence of Social Features Surrounding 2010 Participant Households

Table 7. Prevalence of Social Features Surrounding 2010 Participant Households by Strata of Health Behaviors, Neighborhood Perceptions, and Census Block Group SES and Urbanicity

Table 5. Percent Agreement of Select Items by Domain (Full)

Domain	Features	Items	% Agree	Upper (95% CI)	Lower (95% CI)	Mean
**Neighborhood Characteristics**						
	Positive Aesthetics	Variation in building materials and colors	66.7	65.3	68.2	85.1
		Vegetation	70.5	69.0	71.9	
		Shade trees	72.3	70.9	73.7	
		Street lights	71.3	69.9	72.7	
	Public Amenities	Public trash cans	95.7	95.1	96.3	
		Seating / benches	94.7	94.0	95.4	
		Bike racks	97.1	96.6	97.6	
		Public art	98.5	98.1	98.9	
		Public attractive natural features	95.1	94.4	95.7	
		Neighborhood social / cultural message or event	79.7	78.5	81.0	
		Political message or event	81.2	80.0	82.4	
		Religious message or event	97.2	96.7	97.8	
		Security warning signs	81.9	80.7	83.0	
		Negative Aesthetics				
		Buildings in poor condition	76.4	75.1	77.8	
		Vegetation neglected	70.8	69.3	72.2	
		Careless / harmless litter	53.5	52.0	55.1	
		Hazardous litter	92.2	91.4	93.1	
		Cigarette butts	69.1	67.7	70.6	
		Broken / boarded up windows	98.0	97.6	98.5	
	Advertisements	Fast food advertisements	99.8	99.7	99.9	
		Alcohol advertisements	98.1	97.6	98.5	
		Tobacco advertisements	99.9	99.9	100.0	
		Graffiti	98.2	97.8	98.6	
**Transportation Environment**						
	Transportation	Sidewalks	80.9	79.7	82.1	84.5
		Speed limit	81.9	77.7	86.1	
		Public transportation	87.5	86.4	88.5	
		Pedestrian safety signs (segment)	90.6	89.7	91.5	
		On-street parking with bulb-out (segment)	99.1	98.8	99.4	
		On-street parking without bulb-out (segment)	87.4	86.3	88.4	
		On-street paved, marked bike lane	94.6	93.9	95.3	

		On-street bike lane obstructions	82.3	74.7	89.9	
		On-street bike lane markings worn off	83.5	75.6	91.4	
		Outermost lane wide enough	64.5	62.9	66.1	
		Outermost land obstructions	67.9	65.1	70.8	
		Buffer between street and sidewalk	93.1	92.2	94.0	
		Major misalignments / cracks in sidewalk	85.2	83.9	86.5	
	Street Connectivity	Traffic lanes (segment)	89.8	88.9	90.8	
		Pedestrian crosswalks (segment)	99.5	99.3	99.7	
		Pedestrian crosswalks worn off (segment)	100.0	100.0	100.0	
		Walk / Don't walk signals (segment)	100.0	100.0	100.0	
		Ramps / curb cuts (segment)	99.0	98.6	99.3	
		Medians / pedstrian islands (segment)	97.6	97.1	98.1	
		Pushbutton on Median (segment)	99.9	99.9	100.0	
		Traffic calming devices (segment)	98.7	98.3	99.0	
		Traffic lanes (intersection)	56.3	54.1	58.5	
		Pedestrian crosswalks (intersection)	73.3	71.3	75.2	
		Pedestrian crosswalks worn off (intersection)	91.0	89.8	92.3	
		Intersection type	54.4	49.0	59.7	
		Walk / Don't walk signals (intersection)	91.6	90.4	92.8	
		Ramps / curb cuts (intersection)	48.3	46.1	50.5	
		Pedestrian safety signs (intersection)	75.9	74.0	77.8	
		Medians / pedstrian islands (intersection)	93.7	92.6	94.8	
		Pushbutton on Median (intersection)	94.0	90.4	97.6	
		Traffic calming devices (intersection)	96.9	96.1	97.7	
		Traffic volume (intersection)	46.5	44.3	48.7	
**Destinations and Land Use**						
	Land Use Diversity	Street type	91.4	90.5	92.3	96.5
		Single family homes	85.7	84.6	86.8	

		Multi-unit homes (2-6 units)	79.5	78.2	80.7	
		Apartment building / complex (> 6 units)	92.4	91.6	93.3	
		Apartment over retail	97.0	96.5	97.6	
		Mobile home or trailer park / community	100.0	100.0	100.0	
		Other residential buildings	99.7	99.5	99.8	
		Farm complexes	99.9	99.9	100.0	
		Off-road walking / biking trails or paths	95.3	94.6	95.9	
		Manufacturing facilities	99.4	99.2	99.7	
		Schools	97.7	97.2	98.2	
		Parking lots / garages	93.8	93.0	94.5	
		Parks or designated green spaces	93.5	92.7	94.3	
		Undeveloped land / farmlands / woodlands	95.2	94.6	95.9	
		Railroad (segment)	99.8	99.6	99.9	
		Number of stories of tallest building in segment	72.6	71.1	74.0	
		Type of building (tallest building)	85.1	84.0	86.2	
		Topography	81.0	79.8	82.2	
		Abandoned buildings	99.3	99.0	99.6	
**Non-Residential Destinations**						
	Recreational	Off-road walking / biking trails or paths	95.3	94.6	95.9	
		Schools	97.7	97.2	98.2	
		Indoor fitness facilities	99.9	99.8	100.0	
		Sports / playing fields, courts, or tracks	96.8	96.3	97.4	
		Playgrounds or splash pads	96.5	95.9	97.1	
		Golf courses	100.0	100.0	100.0	
		Pools (indoor or outdoor)	100.0	100.0	100.0	
		Other recreational facilities	98.8	98.5	99.2	
	Alcohol and Liquor Outlets	Bars / night clubs	99.2	98.9	99.5	
		Liquor / tobacco stores	99.5	99.3	99.7	
		Wine stores	99.9	99.8	100.0	
	Restaurants	Fast food restaurants	98.9	98.6	99.2	
		Other restaurants	97.2	96.7	97.8	
		Coffee shops	99.6	99.4	99.8	
		Specialty / ethnic food stores	99.8	99.6	99.9	
	Food Outlets	Food supermarkets or grocery	99.9	99.8	100.0	

		stores				
		Convenience stores or gas station stores	99.8	99.6	99.9	
		Gas stations	99.7	99.6	99.9	
	Health Care	Pharmacies	99.4	99.2	99.6	
		Health care facilities	98.7	98.4	99.1	
	Retail	Retail stores	97.2	96.7	97.8	
		Indoor malls, department stores, or "big box" stores	100.0	100.0	100.0	
		Service providers	93.2	92.4	94.0	
	Fitness	Indoor fitness facilities	99.9	99.8	100.0	
		Cultural entertainment facilities	99.1	98.8	99.4	
		Other entertainment facilities	99.9	99.8	100.0	
	Religious	Non-religious community centers	99.4	99.2	99.6	
		Church, synagogue, mosque, or other religious centers	98.0	97.6	98.5	
		Educational facilities	97.1	96.6	97.6	
		Government facilities	98.9	98.6	99.2	
		Transportation facilities	99.8	99.7	99.9	
		Hotels, motels, or inns	99.0	98.7	99.3	
	Office and Work Space	Office space	98.3	97.9	98.7	
		Warehouses	98.3	97.9	98.7	
**Social Environment**						
	Signs of Social Capital	Neighborhood social / cultural message or event	79.7	78.5	81.0	81.1
		Political message or event	81.2	80.0	82.4	
		Religious message or event	97.2	96.7	97.8	
		Security warning signs	81.9	80.7	83.0	
	Active Engagement	People engaging in other physical activities	85.9	84.8	87.0	
		People walking	67.4	65.9	68.9	
		People biking	75.0	73.6	76.3	

Table 6. Sociodemographic Characteristics and Prevalence of Social Features Surrounding 2010 Participant Households

	Total population (n =939)	Total population	Neighborhood Social or Cultural Message	Security Warnings or Signs	Active Engagement – People walking or Biking	Fitness Centers
**Individual Demographics**	**n**	**(weighted %, 95% CI)**	**(row %, 95% CI, Chi Square)**	**(row %, 95% CI, Chi Square)**	**(row %, 95% CI, Chi Square)**	**(row %, 95% CI. Chi Square)**
**Gender**			0.4090	0.4637	**0.0749**	0.8699
Male	421	50.1 (47.9-52.3)	64.3 (56.5-72.2)	54.6 (46.3-62.9)	68.2 (59.3-77.2)	7.9 (2.9-12.9)
Female	518	49.9 (47.7-52.3)	62.7 (55.2-70.3)	55.9 (47.9-63.9)	71.6 (64.1-79.1)	7.4 (4.3-10.5)
**Age**			**0.0198**	0.7221	**0.0004**	0.2100
21-29	155	19.8 (14.6-24.9)	71.7 (57.8-85.7)	61.4 (44.5-78.2)	83.9 (75.0-92.9)	13.3 (3.2-23.4)
30-39	148	18.0 (14.6-21.5)	75.0 (66.3-83.7)	51.6 (39.6-63.6)	75.4 (66.7-84.0)	6.8 (1.7-11.8)
40-49	204	22.1 (18.5-25.7)	60.0 (48.7-71.4)	55.0 (44.9-65.0)	61.7 (48.0-75.3)	6.1 (2.5-9.7)
50-64	311	29.6 (25.3-33.9)	56.2 (45.6-66.8)	53.2 (43.7-62.8)	64.0 (53.9-74.1)	6.2 (2.1-10.2)
≤ 65	121	10.5 (8.5-12.5)	56.6 (42.6-70.6)	56.4 (44.0-68.8)	68.1 (55.9-80.4)	6.0 (0.2 -11.8)
**Race/Ethnicity**			**0.0002**	**<0.0001**	**0.0001**	0.5862
White (Non-Hispanic)	832	87.5 ((84.6-90.3)	61.2 (53.3-69.1)	52.0 (43.9-60.1)	67.2 (58.6-75.9)	7.3 (4.2-10.4)
Non-white	104	12.5 (9.7-15.4)	80.7 (72.2-89.3)	78.5 (68.4-88.7)	88.4 (80.6-96.2)	10.2 (0.00-22.2)
**Marital status**			**0.0001**	**0.0330**	**<0.0001**	**0.0004**
Married, with partner	611	65.1 (59.4-70.8)	57.9 (49.6-66.3)	51.0 (42.6-59.4)	64.3 (54.6-73.9)	4.6 (1.7-7.5)
Never married	176	21.1 (15.7-26.5)	80.8 (70.3-91.2)	67.1 (53.5-80.8)	85.9 (78.7-93.2)	17.5 (7.7-27.2)
Single (divorced, widowed)	150	13.8 (10.9-16.6)	64.7 (54.3-75.2)	58.3 (45.8-70.7)	72.1 (60.8-83.3)	7.2 (1.3-13.1)
**Education status**			0.0995	0.4692	**0.0204**	0.5843
High School or less	236	24.7 (21.2-28.2)	55.5 (43.8-67.2)	56.8 (47.1-66.5)	61.1 (48.3-73.9)	9.4 (4.2-14.5)
Some college	397	43.1 (39.0-47.3)	64.4 (56.0-72.8)	57.5 (48.1-67.0)	69.7 (60.9-78.5)	7.1 (2.8-11.4)
College or beyond	304	32.2 (27.1-37.4)	69.1 (58.6-79.6)	51.5 (40.5-62.5)	76.8 (67.7-85.8)	7.1 (4.2-10.1)
**Family Income**			0.3681	**0.0458**	**0.0119**	**0.0014**
<100% of FPL	105	12.5 (9.0-15.9)	75.4 (62.9-87.9)	67.7 (54.1-81.2)	82.5 (73.1-91.9)	13.3 (0.9-25.8)
100-199% FPL	144	15.5 (12.1-18.8)	60.5 (46.8-74.2)	58.6 (46.0-71.1)	72.5 (62.2-82.8)	16.2 (6.9-25.5)
200-399% FPL	327	33.5 (29.6-37.5)	60.9 (52.7-69.0)	50.9 (42.0-59.8)	64.4 (54.4-74.3)	4.3 (1.8-6.7)
400%+ FPL	314	33.1 (28.9-37.4)	63.3 (52.0-74.5)	51.3 (41.3-61.2)	68.1 (57.5-78.6)	4.7 (1.9-7.6)
Unknown	49	5.4 (3.8-7.1)	63.2 (44.0-82.4)	68.6 (49.4-87.8)	79.2 (66.6-91.9)	9.0 (0.4-17.6)

Table 7. Prevalence of Social Features Surrounding 2010 Participant Households by Strata of Health Behaviors, Neighborhood Perceptions, and Census Block Group SES and Urbanicity

	Total population (n =939)		Neighborhood Social or Cultural Message	Security Warnings or Signs	Active Engagement – People walking or Biking	Fitness Centers
**Health Promoting Behaviors**	**n**	**(weighted %, 95% CI)**	**(row %, 95% CI, Chi Square)**	**(row %, 95% CI, Chi Square)**	**(row %, 95% CI, Chi Square)**	**(row %, 95% CI. Chi Square)**
**Meet Physical Activity Recommendations (>600 MET/MIN/WEEK)**			0.3651	0.4178	0.7586	**0.0469**
Yes	716	76.6 (73.2-80.0)	62.8 (54.9-70.7)	54.4 (46.5-62.3)	69.7 (61.5-77.9)	6.8 (3.6-10.0)
No	223	23.4 (20.0-26.8)	65.9 (57.7-74.1)	58.0 (47.0-68.9)	70.7 (61.3-80.2)	10.4 (5.9-14.9)
**Servings of Fruits and Vegetables (4-5 daily)**			0.4905	0.6345	0.5296	**0.0287**
Yes	155	17.1 (13.8-20.4)	66.0 (53.2-78.7)	52.7 (41.5-63.8)	71.9 (60.8-83.0)	3.0 (0.2-5.7)
No	683	82.9 (79.6-86.2)	62.0 (53.9-70.1)	55.2 (46.5-63.8)	69.0 (60.2-77.7)	8.4 (4.4-12.4)
**Neighborhood Perceptions**						
**Many destinations within easy walking distance**			**<0.0001**	**0.0077**	**<0.0001**	**0.0200**
Agree	445	58.7 (50.3-67.1)	77.7 (70.3-85.0)	61.3 (50.9-71.7)	87.6 (82.8-92.4)	10.3 (5.3-15.3)
Disagree	391	41.3 (32.9-49.7)	41.4 (32.4-50.4)	45.8 (36.6-54.9)	43.8 (32.0-55.6)	3.5 (0.3-6.7)
**Many interesting things to look at**			**0.0220**	0.9888	**0.0031**	0.3794
Agree	645	78.6 (75.0-82.3)	64.9 (56.7-73.1)	54.8 (46.0-63.7)	72.3 (64.0-80.6)	7.9 (4.4-11.5)
Disagree	190	21.4 ((17.7-25.0)	54.9 (44.2-65.6)	54.8 (44.8-64.7)	59.0 (46.4-71.5)	5.9 (1.3-10.5)
**Community Well Maintained**			0.8769	**0.0211**	**0.0704**	0.8972
Agree	747	89.6 (87.3-92.0)	62.6 (54.2-71.0)	53.4 (45.0-61.8)	68.5 (59.7-77.3)	7.2 (3.9-10.6)
Disagree	84	10.4 (8.0-12.7)	63.5 (51.1-75.9)	68.0 (55.1-80.8)	78.4 (67.1-89.8)	7.7 (0.0-16.2)
**Easy Access to Fresh Fruits and Vegetables**			0.6122	0.4904	0.5188	0.4852
Agree	713	86.6 (83.2-90.0)	63.1 (54.8-71.4)	54.3 (46.0-62.6)	69.9 (61.4-78.5)	7.2 (4.2-10.2)
Disagree	123	13.4 (10.0-16.8)	59.9 (46.7-73.2)	58.5 (44.9-55.1)	66.7 (53.6-79.7)	9.3 (1.1 -17.5)
**Census Block Group Characteristics**						
**Economic Hardship**			**0.0012**	**0.0044**	**0.0037**	**0.0066**
Low	362	39.0 (28.2-49.9)	73.4 (61.7-85.2)	65.0 (54.5-75.5)	80.0 (69.1-90.9)	4.2 (0.7-7.8)
Medium	312	33.1 (21.2-44.9)	45.5 (34.0-57.0)	36.8 (21.4-52.2)	52.3 (36.3-68.4)	4.0 (0.0-8.3)
High	265	27.9 (19.5-36.2)	71.1 (58.8-83.4)	63.5 (49.6-77.5)	76.7 (65.3-88.1)	16.8 (6.2-27.5)
**Urbanicity (Census 2010 urbanized areas & urban clusters)**			**<0.0001**	**<0.0001**	**<0.0001**	**0.0051**
Urban	587	69.2 (60.2-78.1)	79.0 (71.9-86.1)	65.4 (56.4-74.3)	90.0 (84.6-95.4)	10.4 (6.1-14.8)
Rural	352	30.8 (21.9-39.8)	28.9 (17.9-40.0)	32.6 (20.8-44.4)	24.8 (15.0-34.5)	1.4 (0.0-3.7)
